# Nanocapillary sampling coupled to liquid chromatography mass spectrometry delivers single cell drug measurement and lipid fingerprints†

**DOI:** 10.1039/d2an01732f

**Published:** 2023-01-27

**Authors:** Holly-May Lewis, Priyanka Gupta, Kyle D. G. Saunders, Shazneil Briones, Johanna von Gerichten, Paul A. Townsend, Eirini Velliou, Dany J. V. Beste, Olivier Cexus, Roger Webb, Melanie J. Bailey

**Affiliations:** a Department of Chemistry, University of Surrey Guildford UK m.bailey@surrey.ac.uk; b Department of Chemical and Process Engineering, University of Surrey Guildford UK; c Centre for 3D Models of Health and Disease, University College London – Division of Surgery and Interventional Science London UK; d School of Biosciences and Medicine, University of Surrey Guildford UK; e Ion Beam Centre, University of Surrey Guildford UK

## Abstract

This work describes the development of a new approach to measure drug levels and lipid fingerprints in single living mammalian cells. Nanocapillary sampling is an approach that enables the selection and isolation of single living cells under microscope observation. Here, live single cell nanocapillary sampling is coupled to liquid chromatography for the first time. This allows molecular species to be separated prior to ionisation and improves measurement precision of drug analytes. The efficiency of transferring analytes from the sampling capillary into a vial was optimised in this work. The analysis was carried out using standard flow liquid chromatography coupled to widely available mass spectrometry instrumentation, highlighting opportunities for widespread adoption. The method was applied to 30 living cells, revealing cell-to-cell heterogeneity in the uptake of different drug molecules. Using this system, we detected 14–158 lipid features per single cell, revealing the association between bedaquiline uptake and lipid fingerprints.

## Abstract 2

This work describes the development of a new approach to measure drug levels and lipid fingerprints in single living cells.

## Graphical abstract

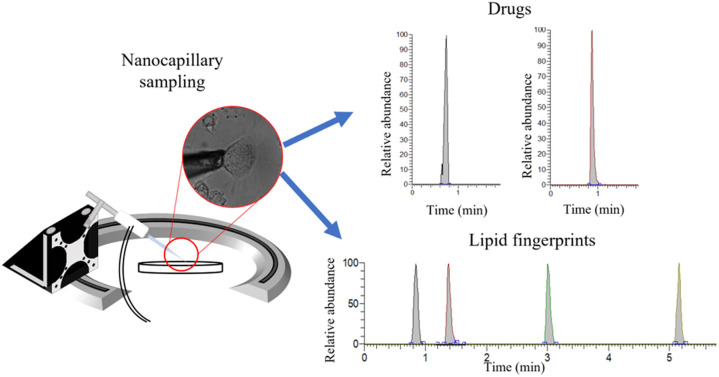

## Introduction

Single cell mass spectrometry has been the subject of a steadily growing number of publications in recent years.^1–17^ This is because bulk measurements of populations of cells does not identify heterogeneity, which is a fundamental property of all biological systems and has wide ranging consequences including for cell to cell communication, treatment of infectious diseases and cancers.^1^ Therefore, analysing single cells is crucial to answering fundamental biological questions and has wide reaching impact including in drug discovery applications.^2^ We must therefore develop sensitive and reliable methods to probe single cells.

Whilst methods for single cell genomics and transcriptomics are well established, single cell mass spectrometry is especially challenging because the analytes cannot be amplified, unlike nucleic acids.^10^ Despite this, mass spectrometry (MS) techniques can offer excellent selectivity to biomolecules without the need for labelling, and there has been some recent success in detecting the low concentrations of analytes in single cells.^11^ For example, developments in single cell proteomics has revealed heterogeneity in cellular protein signatures at an unprecedented level of detail.^9,12–14^ However, mass spectrometry analysis of smaller molecules at the single cell level presents a significant analytical challenge, due to the minute volume of cell content and the wide range of concentrations of metabolites, lipids and proteins in a cell.^15^

The imaging mass spectrometry techniques, matrix assisted laser desorption/ionisation (MALDI) and secondary ionisation mass spectrometry (SIMS), have sufficient spatial resolution to detect metabolites and drugs in single eukaryotic cells.^2–8,10,16–18^ These techniques have been used to show cell to cell heterogeneity,^19,20^ however, since these techniques are direct-MS methods, there is no chromatographic separation of analytes prior to ionisation. This means that analytes are ionised simultaneously, leading to ion suppression, which can limit sensitivity. In addition, in most implementations, MALDI and SIMS are operated under vacuum, precluding the analysis of living cells.^21^

Recently, nanocapillary sampling approaches have been used to extract living cells or intra-cellular contents from 2D culture, which are then analysed using mass sprectrometry.^9,22–40^These approaches, also termed Video-MS, Live Single-Cell MS, or Direct Analyte Probe Nanoextraction (DAPNe) allow a target cell of interest to be extracted into a capillary under video-microscope observation. Nanocapillary sampling approaches can sample single, live cells whilst retaining spatial information and are therefore advantageous. However, the extracted contents are analysed using nanospray ionisation (NSI). In these implementations, analytes are not separated prior to ionisation, which, as for the imaging methods, can lead to ion suppression and matrix effects.^41^

Our work using nanocapillary sampling was successful at quantifying local drug concentrations in tissue samples.^41,42^ We demonstrated that the addition of a chromatography step into the workflow significantly improves measurement precision compared with nanospray ionisation (NSI), with the further advantage of separating lipid classes and allowing the use of automated databases that rely on peak assignment. Here, we advance this work by using nanocapillary sampling coupled to LC-MS to detect and quantify drug molecules (antibiotics) and simultaneously generate a lipid fingerprint from single living cells. We have explored and optimised the factors affecting transfer of drug analytes from the sampling tip into LC-MS vials, their pick-up into a separation system and transport to the mass spectrometer. This approach overcomes several of the disadvantages of using NSI for single cell analysis. Firstly, NSI is not automated, and so there is a burden on the operator to manually change samples (unlike LC-MS autosampling systems); most peak identification software require a chromatographic peak, which cannot be provided by NSI; and importantly, the use of LC-MS substantially improves measurement precision and opens up the possibility of separating isobaric species. The method identified a correlation between lipid fingerprints and drug uptake into cells.

We successfully performed single cell mass spectrometry to measure drugs and lipid fingerprints, which can now be applied to a variety of different mammalian cell types and drugs *ex vivo*. This work will therefore be of significant interest to a range of researchers developing single cell mass spectrometry approaches including those interested in inter-cell lipid and drug uptake/penetration heterogeneity, which is a major impediment to the effective treatment of both cancer and infectious diseases.

## Materials and methods

### Chemicals and reagents

Certified reference materials (>97% purity) of the drug analytes isoniazid, rifampicin, ethambutol, pyrazinamide, and bedaquiline were obtained from Sigma Aldrich. All solvents (methanol (MeOH), ethanol (EtOH), water (H_2_O), isopropanol (IPA), acetonitrile (ACN) and formic acid (FA)) were Optima™ LC-MS grade, obtained from Fischer Scientific.

The culture media for the cell culture procedure was prepared as previously described in Wishart *et al.*^43^ Dulbecco's modified Eagle's medium (DMEM) with high glucose (Sigma-Aldrich, Merck, UK) was supplemented with 10% fetal bovine serum (Fisher Scientific, Loughborough, UK), 1% penicillin/streptomycin (Fisher Scientific, UK), and 2 mM l-glutamine (Sigma-Aldrich, Merck UK).

### Preparation of cell cultures

Human pancreatic adenocarcinoma cells (PANC-1) (ATCC) were seeded at an initial seeding density of 0.5 million cells in a 10 cm dish and cultured for 48–72 hours. Before cell sampling, the DMEM media was replaced with DMEM media spiked with a 100 μM solution of the combined drugs (analytes isoniazid, rifampicin, ethambutol, pyrazinamide, and bedaquiline) (solution at 37 °C) and the flask was then incubated for 24 hours to allow drug uptake. The dosing was based on previous work exploring bedaquiline within cells.^44^ Before nanocapillary extraction, the culture media was removed from the Petri dish and the cells were washed three times with phosphate-buffered saline (PBS) solution, before the addition of 5 ml PBS to the Petri dish. To ensure the concentration of the drugs were not toxic to the cells, a standard trypan blue cell viability assay was performed on the drug dosed cells.^45^

### Nanocapillary sampling settings

**Fig. 1:**
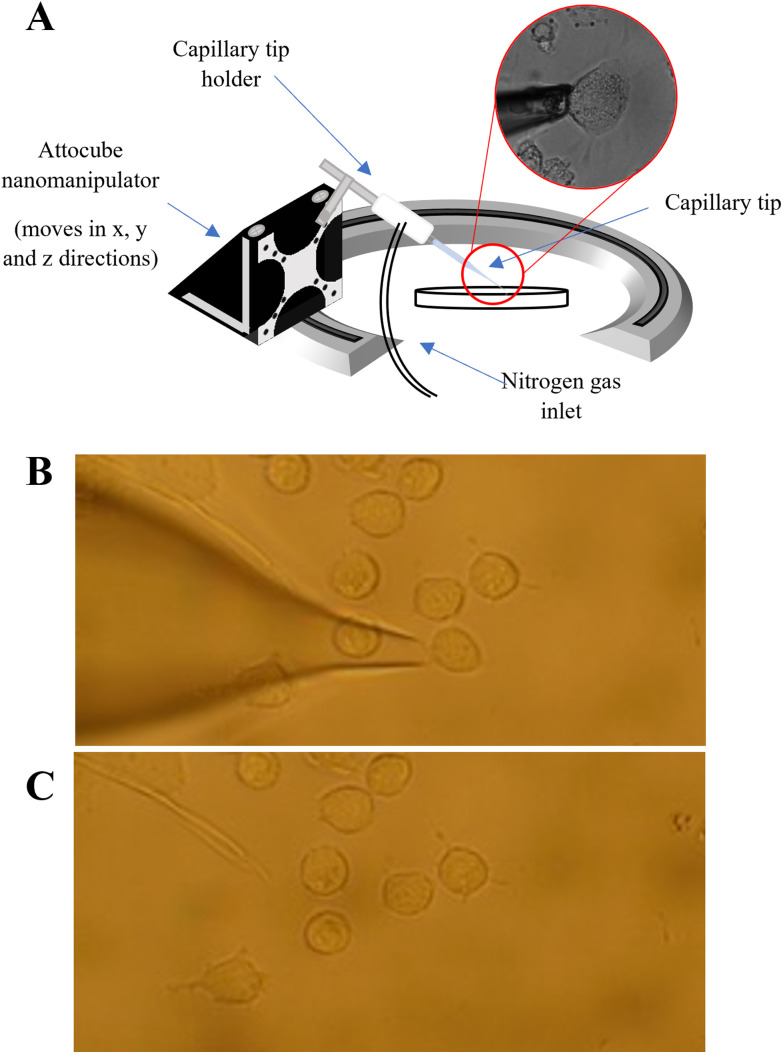
(A): Schematic of the nanocapillary sampling system, in which a nanocapillary is directed to the chosen cell using a nanomanipulator. A back pressure is applied using a pressure injector, to pull the cell into the nanocapillary; microscope images to show the extraction of a single cell using nanocapillary sampling where (B) before the start of an extraction showing that the nanocapillary is positioned at the selected cell and (C) demonstrating that the sampled cell is completed extracted leaving the surrounding cells still in place.

A PUL-1000 tip puller from World Precision Instruments (WPI) was used to pull glass capillary tips (outer diameter 1.2 mm) for cell extraction. This generated capillaries with ∼20 μm diameter, similar in diameter to the PANC-1 cells (20–40 μm in diameter). The pulled tips were inserted into the tip holder of the nanocapillary sampling system Fig. (1A). The cells were located using a Zeiss Axiovert 40C inverted microscope and only similarly sized, adherent cells were sampled. The microscope software AmScope was used to measure the cell diameter of the target cells prior to extraction. The tip was inserted into the capillary tip holder (see Fig. 1A) and was lowered towards a target cell using a nanomanipulator (Attocube), stopping as the end of the tip came into focus. A forward pressure (0.5 psi) was applied to the tip using a PM2000 microinjector (MicroData Instrument, USA) to prevent the PBS solution from being drawn up by capillary action. The target cell was then aspirated into the tip by applying a back (fill) pressure (Fig. 1B and C). The forward pressure, fill pressure and fill time settings were optimised (at 0.5 psi, 5 psi and 0.1s respectively) to ensure that a single cell could be drawn into the tip, whilst minimising the aspiration of PBS. After extraction of a cell, 5 μL of 50 : 50 MeOH/EtOH was added to the back of the tip to lyse the cell. The contents of the tip were then pushed into an LC vial using a gas syringe. This total procedure took ∼10–15 minutes per cell.

### LC-MS settings

The liquid chromatography (LC) method was adapted from Sinclair *et al.*^46^ LC was conducted on a Thermo Scientific Ultimate 3000 UHPLC system. Analytes were separated using an ACQUITY UPLC BEH C18 column (1.7 μm, 2.1 mm × 100 mm), at a flow rate of 0.3 ml min^−1^ at 55 °C. The injection volume was 5 μL, with a needle height of 0.1 mm. This was set to load the entire sample volume onto the column in a single injection. The mobile phases were 60 : 40 acetonitrile/water and 90 : 10 isopropanol/acetonitrile, both with 0.1% formic acid, run in a gradient elution, as shown in Table S1.†

The UHPLC system was coupled to a Thermo Orbitrap Q-Exactive Plus mass spectrometer. The ionisation source was operated with a spray voltage of 3 kV at a capillary temperature of 300 °C. Data was acquired in positive ion mode at a mass range of *m*/*z* 100–1000 using a mass resolution of 70 000 (at *m*/*z* 400) with the automatic gain control (AGC) on and set to 1E6 ions.

### Data analysis

All spectra were analysed using Xcalibur™ (Thermo Fisher Scientific), with drug analytes being identified by their protonated molecular ion peak (within 1 ppm) and retention time compared to certified reference materials. LipidSearch™ (Thermo Fisher Scientific) was used to make lipid peak assignments in the single cells. Only peaks above a 1E4 intensity threshold and within 5 ppm of the calculated *m*/*z* value were selected. The data was corrected using blank samples extracted from identical PBS solution as the surrounding cells using the pressure injector with the same settings as used for cell extraction (diluted with 50 : 50 MeOH/EtOH – blank correction of 10× signal to noise ratio).

To compare different cell populations, MetaboAnalyst 5.0 (MKS Umetrics) was used to conduct partial least squared discriminant analysis (PLS-DA) analysis. Prior to analysis, the data was pareto scaled. The PLS-DA analysis provided a list of lipid features and their corresponding variable importance in projection (VIP). A VIP score is a measure of a variable's importance in the PLS-DA model. It gives the contribution each lipid feature makes to the model, therefore the higher the VIP score, the more it contributes to the model.

### LC-MS method validation

Certified reference materials of each drug were prepared at concentrations 0, 0.1, 0.5, 1, 2, 5, 10, and 20 ng ml^−1^ by diluting the 1 mg ml^−1^ stock solutions in 50 : 50 MeOH/EtOH. 100 μL was aliquoted into glass LC-MS vials with 300 μL inserts (Supelco, UK). 5 μL of each standard was injected onto the LC and 5 replicates of each concentration were measured.

A feature of nanocapillary sampling is that the cell is collected and lysed in a few microlitres of solvent. To characterise the impact of low sample volume on standard flow LC-MS measurement precision and sensitivity, we compared 5 μL injections of 100 ng ml^−1^ drug standard from (A) 5 μL starting volume to (B) 100 μL starting volume, in quintuplicate.

### Comparison of LC-MS and nanospray ionisation (NSI) precision

The precision of the LC-MS method was compared to NSI, the approach usually used in conjunction with nanocapillary sampling. This was done using drug standards, which were diluted to 100 ng ml^−1^ in 50 : 50 MeOH/EtOH. For NSI, 5 μL of the standards were added directly to nanospray emitter tips using a gas syringe, and sprayed directly into the mass spectrometer, using resolution settings of 280 000, 140 000, 70 000 and 35 000. This was conducted in quintuplicate and compared to the LC-MS method described above.

### Optimisation of transfer efficiency

To evaluate the efficiency of transferring samples from the capillary tip to the LC-MS vial, 100 ng ml^−1^ solutions of drugs were prepared in 50 : 50 MeOH/EtOH. 2 μL of the solution was inserted into the back of 5 separate pulled tips and the contents were pushed into 5 separate LC vials; this was repeated for each of the transfer methods outlined below. For comparison, 2 μL of the same solution was pipetted directly into 5 separate LC vials as a reference sample. In each case the solutions were made up to a final volume of 5 μL by addition of mobile phase.

The following methods were tested for transferring samples from the tip into the liquid chromatography vial: (a) “pressure injector” (use of the pressure injector to push analytes from the tip into the vial); (b) “gas syringe” where (use of a gas syringe to push analytes from the tip into the vial); (c) “methanol evaporation”, where method (b) is followed by the addition of 300 μL of methanol to the vial to recover analytes from the walls of the vial. The methanol was allowed to evaporate to dryness, using a gentle stream of nitrogen, and the sample was reconstituted in 5 μL mobile phase; and (d) “backfilling tip and gas syringe” (the tip is backfilled with 3 μL mobile phase and analytes transferred to the vial using the gas syringe).

### Comparison of bulk and single cell LC-MS measurement

To prepare cells for bulk measurement, approximately 10^6^ adhered PANC-1 cells were washed in Dulbecco's phosphate buffer (PBS) three times to remove cell culture media and then after ensuring all the media was removed, 1 mL of ice-cold optima grade water was added. The dish containing the cells was then sealed and lowered into liquid nitrogen for 10 seconds, followed by cell scraping to lift adherent cells. 200 μL was transferred to a 2 mL Eppendorf microcentrifuge tube. The aliquot was freeze–thawed between liquid nitrogen and a water bath set to 37 °C twice before sonicating on ice for 30 seconds. 1.2 mL ice cold MeOH/EtOH (50 : 50 v/v) was added and vortexed for 2 minutes. The aliquot was then centrifuged for 5 minutes at 1000*g*. 200 μL of the supernatant was taken and dried under nitrogen. The solution was then reconstituted to 100 μL on the day of analysis in MeOH/EtOH (50 : 50 v/v/).

### Staining of lipid droplets

PANC-1 cells were harvested from the culture dish by removing the culture media and washing once with PBS before detaching by incubating with Trypsin-EDTA (Sigma-Aldrich, Merch UK). Once ≥90% of cells were detached, cells were washed once with PBS and stained with 5uM BODIPY 493/503 (#D3922, Invitrogen, USA) in PBS and incubated in the dark for 15 minutes at 37 °C with 5% CO_2_. After incubation, cells were washed and resuspended with PBS with 5% FBS before being analysed using a BD FACSCelesta™ Cell Analyser. Data analysis was performed using FlowJo (BD Life Sciences, UK). The experiments were biological replicates of *n* = 3.

## Results and discussion

### Sensitivity and precision of the LC-MS method in detecting low volumes of drug

**Fig. 2:**
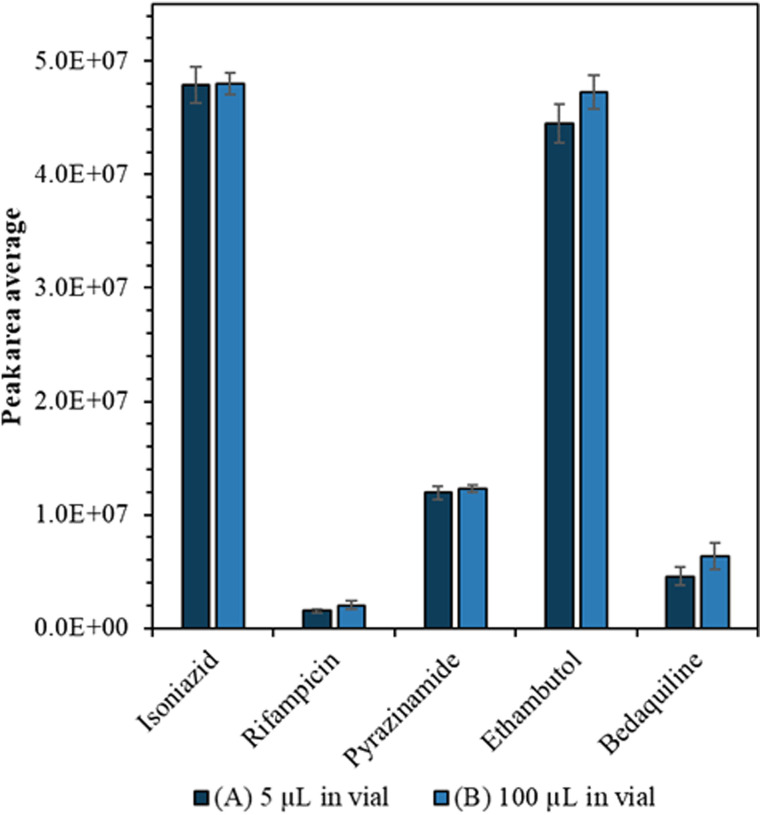
Average peak area measured by LC-MS for 100 ng mL^−1^ drug analytes for a starting volume of (A) 5 μL (dark blue) (B) 100 μL (light blue); using a 5 μL injection volume. The error bars show the standard deviation between the repeat measurements from different vials (*n* = 5).

Drug calibration curves are shown in Fig. S1† and the limits of detection were calculated (Table S2†) using infinite dilutions. Importantly we showed that we could detect the drugs from a small volume without compromising sensitivity or precision. Firstly, we tried to carry the drug analytes in a lower volume of solvent (1, 2, 3, 4 and 5 μL) but found that when the sample volume is less than 5 μL, there was a loss of sensitivity (see Fig. S2†). Furthermore, when we compared the average peak areas of the drugs from (A) 5 μL to (B) 100 μL starting volume there was no significant difference (confirmed by Mann Whitney *U* test, *p* > 0.05) as can be seen in Fig. 2.

We also evaluated whether the LC-MS method exhibited improved precision compared with NSI, which has typically been used for single cell mass spectrometry. Fig. S3† shows that for NSI, there is a trade-off between mass resolution and precision to the anti-TB drug analytes. The precision of the LC-MS method (<10% RSD) is considerably better than NSI (15–55% RSD) regardless of the mass resolution setting.

### Transfer efficiency from tip to LC vial

**Fig. 3:**
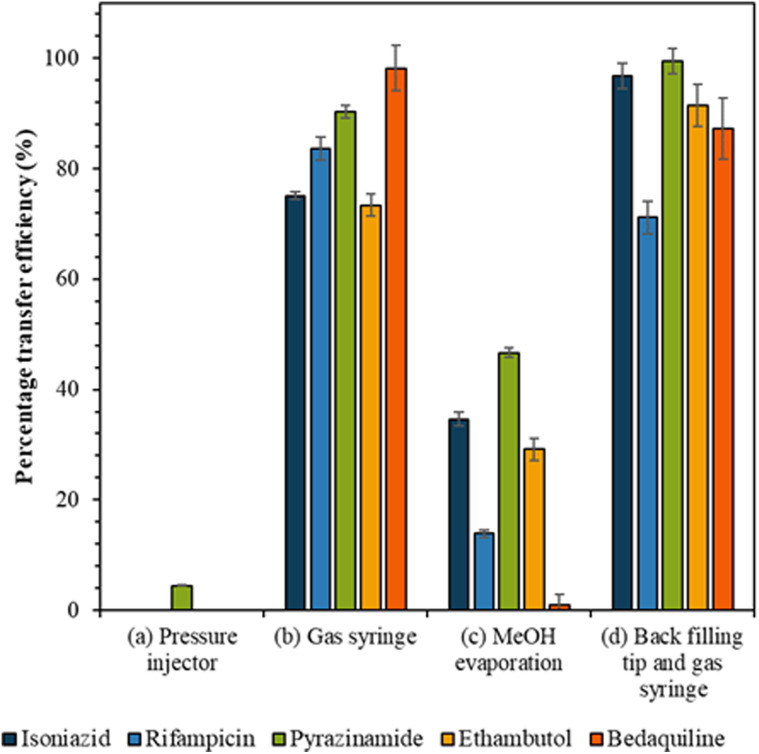
The transfer efficiency (%) for each drug analyte with fixed concentration when transferred from the capillary tip to the LC vial by the following methods: (a) pressure injector, (b) gas syringe, (c) MeOH evaporation and (d) back filling tip and gas syringe, as shown in the method section and with error bars to show the standard deviation between repeats (*n* = 5).

The efficiency of the various approaches to transfer cell extracts from the capillary tip into the vial was calculated by comparison to the reference sample peak intensity (Fig. 3). Use of the pressure injector to elute analytes from the tip resulted in poor transfer efficiency, presumably because in this configuration the tip did not reach the bottom of the vial and analytes were deposited on the walls of the vial. Similarly, the methanol evaporation method resulted in a significant loss of analytes, presumably due to deposition on the walls of the vial as the methanol evaporated. Transfer of analytes *via* gas syringe and particularly after backfilling the tip (method (d)) gave the best recovery (Fig. 3) for the drugs isoniazid, pyrazinamide, and ethambutol (confirmed by Mann Whitney *U* test, *p* > 0.05).

### Anti-TB drug detection in single mammalian cells

We selected a treatment of 100 μM of drugs for 24 h as our conditions for method development and demonstrated high cell viability using a standard assay based on Strober *et al.*^45^ (96.6% remained viable). A total of 30 live single cells were extracted and analysed using our LC-MS method. The microscope images, corresponding diameters (20 to 56 μm) and areas of the cells prior to extraction are shown in Fig. S4.†

**Fig. 4:**
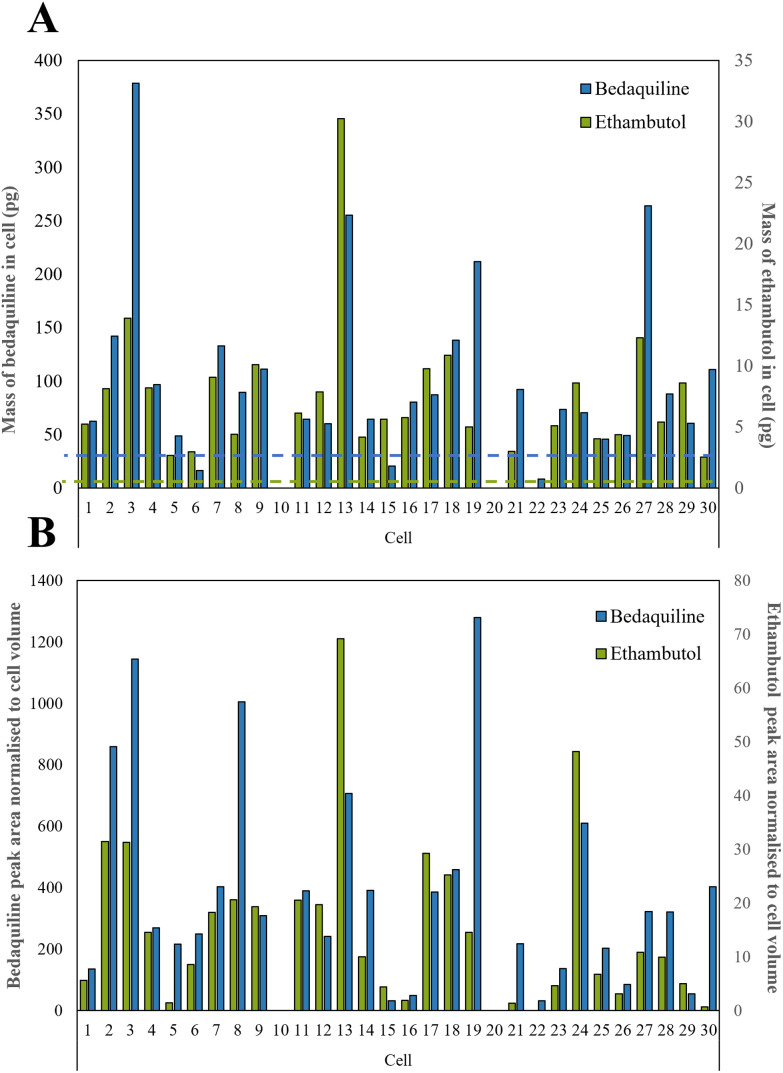
(A) Calculated mass of bedaquiline and ethambutol as pg per single cell with the dashed line showing the LoD for bedaquiline (blue) and ethambutol (green) which were both 2.5 pg respectively as shown in Table S2,† and (B) bedaquiline and ethambutol peak area were normalised to cell volume to take into account the different sizes of cells.

Ethambutol and bedaquiline were detected in 28/30 of the cells (see Fig. S5† for example extracted ion chromatograms as well as a total ion chromatogram (TIC) of a single cell mass spectrum). Pyrazinamide, isoniazid, and rifampicin were not detected. In Fig. 4A, the ethambutol and bedaquiline peak areas were normalised to the calculated cell volume (assuming a spherical cell geometry). Even after normalisation, there was a large variation (97% and 87% for ethambutol and bedaquiline respectively) in the measurement of drug per unit volume at the single cell level, identifying significant heterogeneity in drug uptake by the cells. This variation is not due to either the precision of the analyte transfer and LC-MS method, which was below 20% for all analytes.

In Fig. 4B, the calibration lines were used to quantify the amount of each drug in pg per cell. Bedaquiline was at 8–380 pg per cell and ethambutol lower, at 3–30 pg per cell. For most of the cells, the mass per cell of bedaquiline and ethambutol surpassed the corresponding limit of detection (5–10 pg) of the other (undetected) drugs indicating preferential uptake of bedaquiline and ethambutol by these cells. This is in accordance with other studies showing that bedaquiline and ethambutol are distributed rapidly and accumulate in many cell types and tissues.^47–49^ Ethambutol is distributed rapidly and at higher concentrations in cells and tissues than corresponding plasma^49^ and bedaquiline is known to have amphiphilic properties, so effectively diffuses through the cell membrane.^48^

### Lipid detection in single cells

**Fig. 5:**
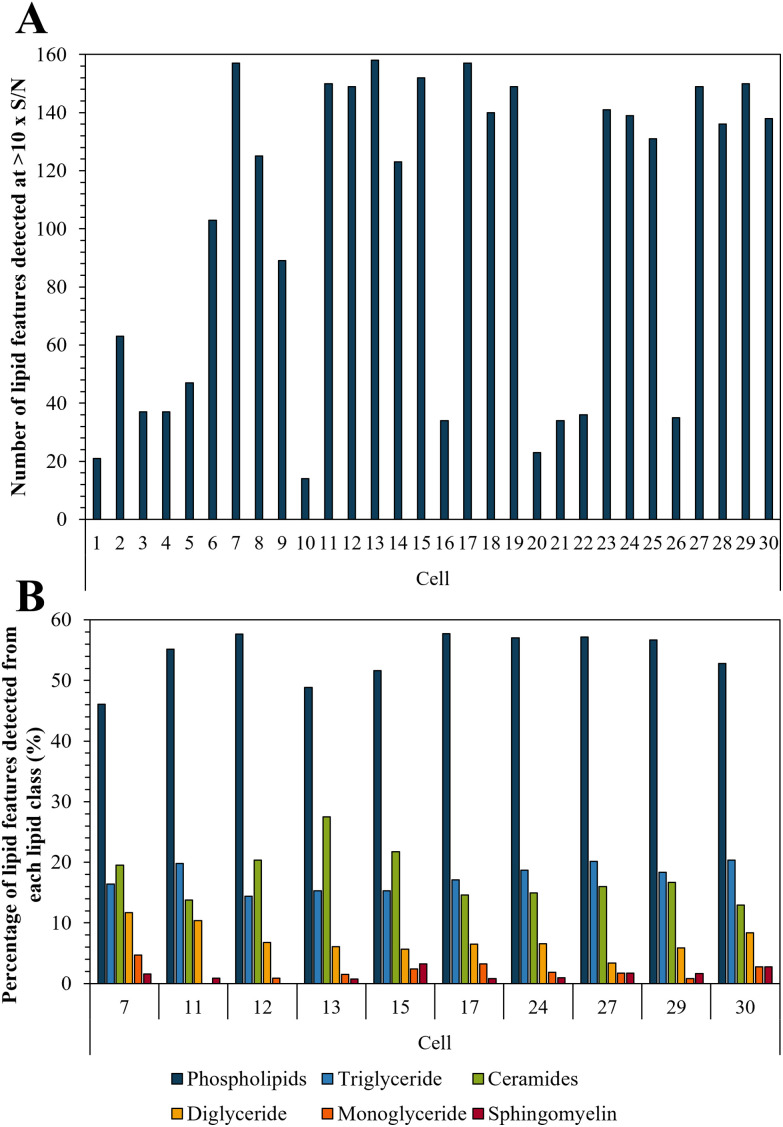
(A) Number of lipid features detected per single cell at >10× S/N; and (B) illustration of lipid fingerprints for the 10 cells with the highest number of detected lipid features.

Bedaquiline has been shown to accumulate in fat laden cells such as foamy macrophages which correlates with the drugs ability to bind to phospholipids.^47,50^ Here we show that such droplets are ubiquitous in PANC-1 cells (Fig. S6†) and therefore we tested whether this impacts on bedaquiline uptake. To do this we simultaneously compared the level of each drug with their lipid fingerprint. Measurement of the LipidSplash standard (Fig. S7†) demonstrated that the LC-MS method was able to provide some separation of lipid classes, and (Fig. S5†) that they did not co-elute with the drug analytes, highlighting an advantage of this approach. The number of lipid features detected per cell ranged from 14 to 158 (Fig. 5A). The lipid features identified by the LipidSearch software for each cell, and their corresponding peak areas have been added to the ESI.† Interestingly, for cell 10 and 20, where no anti-TB drugs were detected, the number of detected lipid features is also correspondingly low.

The ten cells for which the highest number of lipid features were detected were selected to illustrate the lipid fingerprint at the single cell level (Fig. 5B) demonstrating that peaks assigned to phospholipids were the most commonly detected. This is in accordance with previous research as phospholipids are by far the most abundant lipids in eukaryotic cell membranes.^51^ The high proportion of phospholipids in PANC-1 cells has also been reported in other studies, which also see similar lipid features as we have assigned here.^52,53^ Fig. S8† demonstrates the similarity of lipid profiles generated from single cells and bulk extraction. Fig. 5 shows that differences were observed in lipid fingerprints between the single cells, indicative of cell-to-cell heterogeneity.

**Fig. 6:**
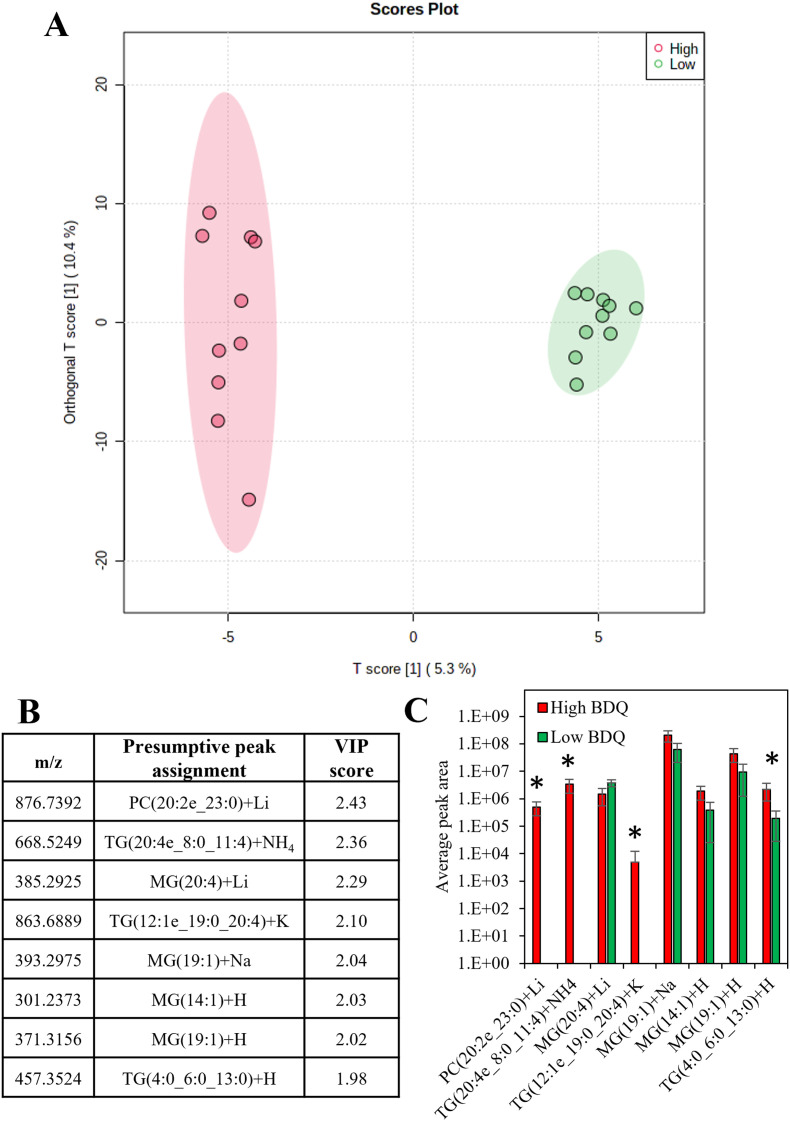
(A) Orthogonal projections to latent structures discriminant analysis (OPLS-DA) for the 10 cells with the highest measured bedaquiline content and 10 cells with the lowest measured bedaquiline content (with 95% confidence interval ellipses), (B) top variable importance in projection scores for the *m*/*z* detected and the presumptive peak assignment, (C) the average peak areas of the top VIP scores for high and low bedaquiline concentrations (those significantly different are labelled).

There was a moderate correlation between the total measured lipid peak intensity per cell and the bedaquiline peak intensity, with an *R*^2^ value of 0.62 (Fig. S9†). Supervised analysis was used to screen for differences between drug uptake and lipid composition using data from cells with the highest and lowest amount of bedaquiline (10 cells from each group were selected). In Fig. 6A, an Orthogonal Projections to Latent Structures Discriminant Analysis (OPLS-DA) model is plotted. The top variable importance in projection (VIP) scores for the detected *m*/*z* values (and presumptive peak assignment) are listed in Fig. 6B and the corresponding peak intensities are plotted in Fig. 6C. The data in Fig. 6 suggests an association between bedaquiline uptake and peaks assigned to glycerolipids. This observation is consistent with the detection of lipid droplets in PANC-1 cells (Fig. S6†) and as previously reported.^54^ For comparison, equivalent analysis was also undertaken for ethambutol (Fig. S10†).

The data here shows that with careful control of the transfer steps, single live cells can be transported from a sampling capillary onto a liquid chromatography column. To maximise recovery, we make the following recommendations: (a) to transfer analytes from the capillary to the vial, the end of capillary should be positioned as close to the bottom of the vial as possible, using a low forward pressure to avoid deposition of analytes on the walls of the vial; (b) to use an insert vial with a convex base, and a low needle height to enable the entire volume of sample to be injected onto the LC.

Nanocapillary sampling followed by LC-MS can be used to quantify drugs, as well as generating lipid fingerprints from single living cells. We have demonstrated the precision of the downstream analysis method and the transfer step to be <20% using certified reference materials, demonstrating that the method is significantly better than nanospray ionisation.^42^ This is sufficient to monitor cell to cell variation in drug uptake, which in this case varied by 87–97%. An additional advantage of using LC-MS is the possibility to separate analytes prior to analysis, reducing ion suppression. This work provides an insight into factors which may contribute to drug uptake and cell penetration, by associating drug uptake with lipid fingerprints.

This approach opens up the possibility to make presumptive lipid peak assignment through their characteristic retention time as well as accurate mass.^55^ Therefore, nanocapillary sampling followed by LC-MS opens the possibility to resolve isobaric species through retention time, which cannot be done with NSI. Although isobaric species were not separated using this method, a different LC-MS method could be applied to achieve this.

### Limitations and future work

The manual sampling method used here needs 10 minutes per cell and requires a skilled user, which limits applications where large numbers of cells are required for statistical robustness. However, a commercial high throughput automated system is in development with the potential to overcome these limitations^56^ which will benefit from these findings. In this work, we made the assumption that cells were fully lysed in the tip by the addition of organic solvent and future work should consider approaches to assess whether the cell is fully lysed, to maximise sensitivity. Using nanoflow chromatography as an alternative to the standard flow method used here could also increase the sensitivity of this approach. However, this would substantially reduce the throughput of measurements, and so for now, users of single cell mass spectrometry must choose between sensitivity, selectivity, and throughput.

The volume of a single cell is only sufficient for one injection into the LC and therefore users need to choose between (A) polarity switching for maximum coverage of lipid features and (B) fixed polarity and maximised sensitivity. For the same reason, we did not generate MS2 data from single cells; lipids are assigned based on their accurate mass and are only presumptive assignments. Future work should explore whether MS/MS data can be used to annotate peaks in single cells, using alignment of retention time and accurate mass.

## Conclusion

We have shown how nanocapillary sampling followed by LC-MS can measure drugs and lipid fingerprints in single living cells. The transfer of analytes from the sampling capillary into the LC-MS vial, and into the LC-MS has been optimised, to ensure sensitive and precise measurement of analytes from low volumes. This has been applied to reveal cell to cell heterogeneity in drug uptake and lipid profiles. We show that bedaquiline uptake was associated with peaks assigned to glycerolipids in PANC-1 cells. This is supported by previous work showing that bedaquiline migrates to lipid droplets, which were also observed in this work. This work provides an approach that can be applied to a variety of mammalian cell types to identify the effects of heterogeneity of lipid species on intracellular drug penetration.

## Author contributions

Conceptualization – H.-M. L., R. W., M. J. B.; methodology – H.-M. L., D. J. V. B., O. C., M. J. B.; validation – K. D. G. S.; formal analysis – H.-M. L., K. D. G. S.; investigation – H.-M. L., P. G., K. D. G. S., S. B.; resources – P. A. T., E. V., D. J. V. B., O. C., R. W., M. J. B.; writing – original draft – H.-M. L., D. J. V. B., O. C., M. J. B.; writing – review & editing – H.-M. L., J. v. G., D. J. V. B., O. C., M. J. B.; visualization – H.-M. L., K. D. G. S., S. B; supervision – P. A. T., E. V., D. J. V. B., O. C., R. W., M. J. B.; funding acquisition – E. V., O. C., R. W., M. J. B.

## Conflicts of interest

There are no conflicts to declare.

## Supplementary Material

AN-148-D2AN01732F-s001

AN-148-D2AN01732F-s002

## References

[cit1] De Silva I. W., Kretsch A. R., Lewis H.-M., Bailey M., Verbeck G. F. (2019). Analyst.

[cit2] Taylor M. J., Lukowski J. K., Anderton C. R. (2021). J. Am. Soc. Mass Spectrom..

[cit3] Huang L., Chen Y., Weng L. T., Leung M., Xing X., Fan Z., Wu H. (2016). Anal. Chem..

[cit4] Passarelli M. K., Pirkl A., Moellers R., Grinfeld D., Kollmer F., Havelund R., Newman C. F., Marshall P. S., Arlinghaus H., Alexander M. R., West A., Horning S., Niehuis E., Makarov A., Dollery C. T., Gilmore I. S. (2017). Nat. Methods.

[cit5] Xiong C., Zhou X., He Q., Huang X., Wang J., Peng W. P., Chang H. C., Nie Z. (2016). Anal. Chem..

[cit6] Amantonico A., Urban P. L., Fagerer S. R., Balabin R. M., Zenobi R. (2010). Anal. Chem..

[cit7] Rubakhin S. S., Sweedler J. V. (2008). Anal. Chem..

[cit8] Passarelli M. K., Ewing A. G., Winograd N. (2013). Anal. Chem..

[cit9] Johnson K. R., Gao Y., Greguš M., Ivanov A. R. (2022). Anal. Chem..

[cit10] Oikawa A., Saito K. (2012). Plant J..

[cit11] Yin L., Zhang Z., Liu Y., Gao Y., Gu J. (2019). Analyst.

[cit12] Petelski A. A., Emmott E., Leduc A., Huffman R. G., Specht H., Perlman D. H., Slavov N. (2021). Nat. Protoc..

[cit13] Specht H., Emmott E., Petelski A. A., Huffman R. G., Perlman D. H., Serra M., Kharchenko P., Koller A., Slavov N. (2021). Genome Biol..

[cit14] Schoof E. M., Furtwängler B., Üresin N., Rapin N., Savickas S., Gentil C., Lechman E., auf dem Keller U., Dick J. E., Porse B. T. (2021). Nat. Commun..

[cit15] Dittrich P. S., Armbrecht L. (2017). Anal. Chem..

[cit16] Dueñas M. E., Essner J. J., Lee Y. J. (2017). Sci. Rep..

[cit17] Szakal C., Narayan K., Fu J., Lefman J., Subramaniam S. (2011). Anal. Chem..

[cit18] Bhaduri A., Neumann E. K., Kriegstein A. R., Sweedler J. V. (2021). JACS Au.

[cit19] Zhang Y., Guillermier C., De Raedt T., Cox A. G., Maertens O., Yimlamai D., Lun M., Whitney A., Maas R. L., Goessling W., Cichowski K., Steinhauser M. L. (2020). iScience.

[cit20] Yang B., Patterson N. H., Tsui T., Caprioli R. M., Norris J. L. (2018). J. Am. Soc. Mass Spectrom..

[cit21] Chughtai K., Heeren R. M. A. (2010). Chem. Rev..

[cit22] Masujima T. (2009). Anal. Sci..

[cit23] Kajiyama S., Harada K., Fukusaki E., Kobayashi A. (2006). J. Biosci. Bioeng..

[cit24] Marakalala M. J., Raju R. M., Sharma K., Zhang Y. J., Eugenin E. A., Prideaux B., Daudelin I. B., Chen P. Y., Booty M. G., Kim J. H., Eum S. Y., Via L. E., Behar S. M., Barry C. E., Mann M., Dartois V., Rubin E. J. (2016). Nat. Med..

[cit25] Li Z., Cheng S., Lin Q., Cao W., Yang J., Zhang M., Shen A., Zhang W., Xia Y., Ma X., Ouyang Z. (2021). Nat. Commun..

[cit26] Zhang L., Khattar N., Kemenes I., Kemenes G., Zrinyi Z., Pirger Z., Vertes A. (2018). Sci. Rep..

[cit27] Hamilton J. S., Verbeck G. F. (2016). J. Anal. Oncol..

[cit28] Hamilton J. S., Aguilar R., Petros R. A., Verbeck G. F. (2017). J. Am. Soc. Mass Spectrom..

[cit29] Phelps M. S., Verbeck G. S. (2015). Anal. Methods.

[cit30] Phelps M. S., Sturtevant D., Chapman K. D., Verbeck G. F. (2016). J. Am. Soc. Mass Spectrom..

[cit31] Phelps M. S., Hamilton J. S., Verbeck G. F. (2014). Rev. Sci. Instrum..

[cit32] Masujima T. (1999). Anal. Chim. Acta.

[cit33] Ali A., Abouleila Y., Shimizu Y., Hiyama E., Watanabe T. M., Yanagida T., Germond A. (2019). Anal. Chem..

[cit34] Date S., Mizuno H., Tsuyama N., Harada T., Masujima T. (2012). Anal. Sci..

[cit35] Tsuyama N., Mizuno H., Tokunaga E., Masujima T. (2008). Anal. Sci..

[cit36] Abouleila Y., Onidani K., Ali A., Shoji H., Kawai T., Lim C. T., Kumar V., Okaya S., Kato K., Hiyama E., Yanagida T., Masujima T., Shimizu Y., Honda K. (2019). Cancer Sci..

[cit37] Pan N., Rao W., Kothapalli N. R., Liu R., Burgett A. W. G., Yang Z. (2014). Anal. Chem..

[cit38] Pan N., Rao W., Yang Z., Yu K. (2015). LCGC North Am..

[cit39] Newman C. F., Havelund R., Passarelli M. K., Marshall P. S., Francis I., West A., Alexander M. R., Gilmore I. S., Dollery C. T. (2017). Anal. Chem..

[cit40] Yuan F., Zhang D.-W., Liu J.-X., Zhou Y.-L., Zhang X.-X. (2016). Analyst.

[cit41] de Jesus J., Bunch J., Verbeck G., Webb R. P., Costa C., Goodwin R. J. A., Bailey M. J. (2018). Anal. Chem..

[cit42] Lewis H.-M., Webb R. P., Verbeck G. F., Bunch J., De Jesus J., Costa C., Palitsin V., Swales J., Goodwin R. J. A., Sears P., Bailey M. J. (2019). Anal. Chem..

[cit43] Wishart G., Gupta P., Nisbet A., Schettino G., Velliou E. (2021). Cancers.

[cit44] Preiss L. , Langer J. D., Yildiz Ö., Eckhardt-strelau L., Guillemont J. E. G., Koul A. and Meier T., Sci. Adv., 2015 10.1126/sciadv.1500106 PMC4640650 26601184

[cit45] Strober W. (2019). Curr. Protoc. Immunol..

[cit46] Sinclair E. , Trivedi D. K., Sarkar D., Walton-Doyle C., Milne J., Kunath T., Rijs A. M., de Bie R. M. A., Goodacre R., Silverdale M. and Barran P., Nat. Commun., 2021, 12 1, 1592 33707447 10.1038/s41467-021-21669-4 PMC7952564

[cit47] van Heeswijk R. P. G., Dannemann B., Hoetelmans R. M. W. (2014). J. Antimicrob. Chemother..

[cit48] Svensson E. M., du Bois J., Kitshoff R., de Jager V. R., Wiesner L., Norman J., Nachman S., Smith B., Diacon A. H., Hesseling A. C., Garcia-Prats A. J. (2018). Br. J. Clin. Pharmacol..

[cit49] Liss R. H., Letourneau R. J., Schepis J. P. (1981). Am. Rev. Respir. Dis..

[cit50] Fearns A., Greenwood D. J., Rodgers A., Jiang H., Gutierrez M. G. (2020). PLoS Biol..

[cit51] Alberts B. , Johnson A., Lewis J., Raff M., Roberts K. and Walter P., Molecular Biology of the Cell, Garland Science, New York, 4th edn., 2002

[cit52] Liu Q., Ge W., Wang T., Lan J., Martínez-Jarquín S., Wolfrum C., Stoffel M., Zenobi R. (2021). Angew. Chem., Int. Ed..

[cit53] Zhang H., Wang Y., Guan L., Chen Y., Chen P., Sun J., Gonzalez F. J., Huang M., Bi H. (2021). J. Pharm. Anal..

[cit54] Sunami Y., Rebelo A., Kleeff J. (2018). Cancers.

[cit55] Vu N., Narvaez-Rivas M., Chen G. Y., Rewers M. J., Zhang Q. (2019). Anal. Bioanal. Chem..

[cit56] Yokogawa Electric Corporation , Single Cellome™ System 2000, https://www.yokogawa.com/uk/solutions/products-platforms/life-science/single-cellome/ss2000/

