# Supplementary material for: Nanocapillary sampling coupled to liquid chromatography mass spectrometry delivers single cell drug measurement and lipid fingerprints
Source: Analyst. 2023 Jan 27;148(5):1041–9. doi: 10.1039/d2an01732f (PMC9969958; doi:10.1039/d2an01732f)
Supplement: AN-148-D2AN01732F-s002 [file AN-148-D2AN01732F-s002.pdf]

Electronic Supplementary Material (ESI) for Analyst.
This journal is © The Royal Society of Chemistry 2023

Supporting information

Nanocapillary sampling coupled to liquid chromatography mass spectrometry delivers single cell drug measurement and lipid fingerprints

Holly-May Lewis[1], Priyanka Gupta[2,3], Kyle D.G. Saunders[1] Shazneil Briones[4], Johanna von Gerichten[1], Paul A. Townsend[4], Eirini Velliou[2,3], Dany J.V. Beste[4], Olivier Cexus[4], Roger Webb[5] and Melanie J. Bailey[1*]

[1] Department of Chemistry, University of Surrey, Guildford, UK

[2] Department of Chemical and Process Engineering, University of Surrey, Guildford, UK

[3] Centre for 3D Models of Health and Disease, University College London - Division of Surgery and Interventional Science, London, UK

[4] School of Biosciences and Medicine, University of Surrey, Guildford, UK

[5] Ion Beam Centre, University of Surrey, Guildford, UK

*Email: m.bailey@surrey.ac.uk

Table S1: The liquid chromatography gradient applied in this work, where mobile phase solvent A) 60:40 acetonitrile/water + 0.1% formic acid and mobile phase solvent B) 90:10 isopropanol/acetonitrile + 0.1% formic acid

Time (min)	% Solvent ***A***	% Solvent ***B***
0.0	60	40
1.0	50	50
3.6	31	69
12.0	12	88
14.0	60	40
16.0	60	40

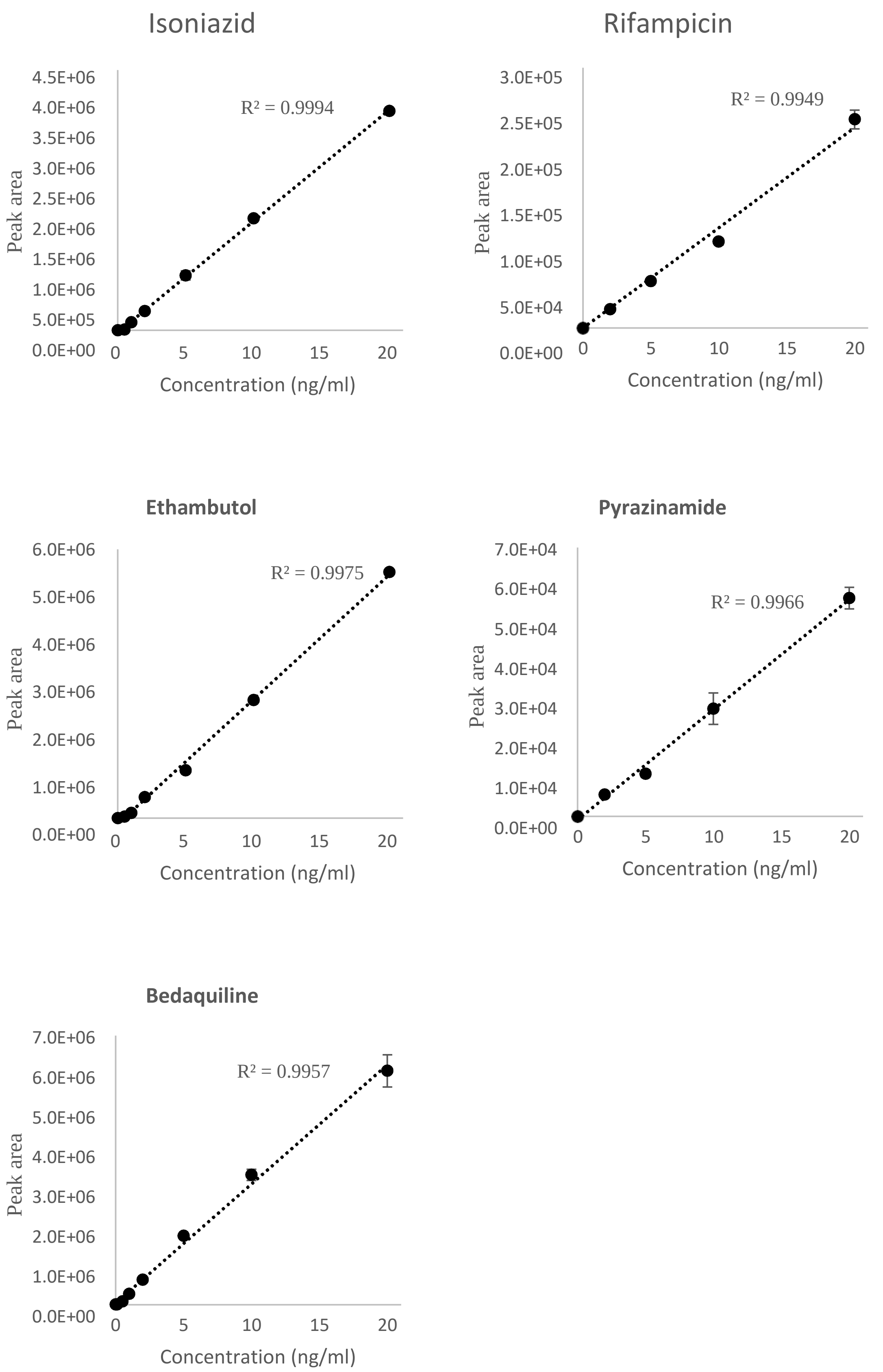

Figure S1: Calibration curves for isoniazid, rifampicin, ethambutol, pyrazinamide and bedaquiline and concentrations 0, 0.1, 0.5, 1, 2, 5, 10, and 20 ng/ml diluted in MeOH:EtOH using LC-MS method; (n=5) injections per sample where the error bars show the standard deviation between repeats.

Table S2. Limits of detection for the drug analytes in solution, determined by a method of infinite dilutions.

	Limit of detection (ng/ml)	Limit of detection (pg)
Isoniazid	1	5
Rifampicin	2	10
Ethambutol	0.5	2.5
Pyrazinamide	2	10
Bedaquiline	0.5	2.5

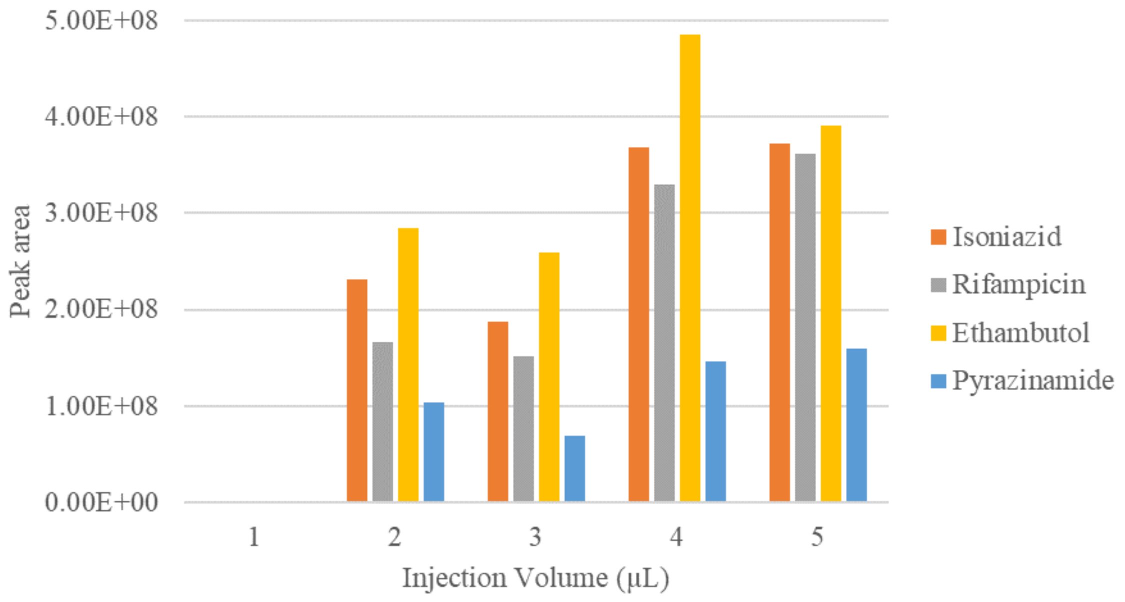

Figure S2: Signal intensity for isoniazid, rifampicin, ethambutol and pyrazinamide where the same mass (10 ng) of analyte is diluted in different injection volumes (n=1)

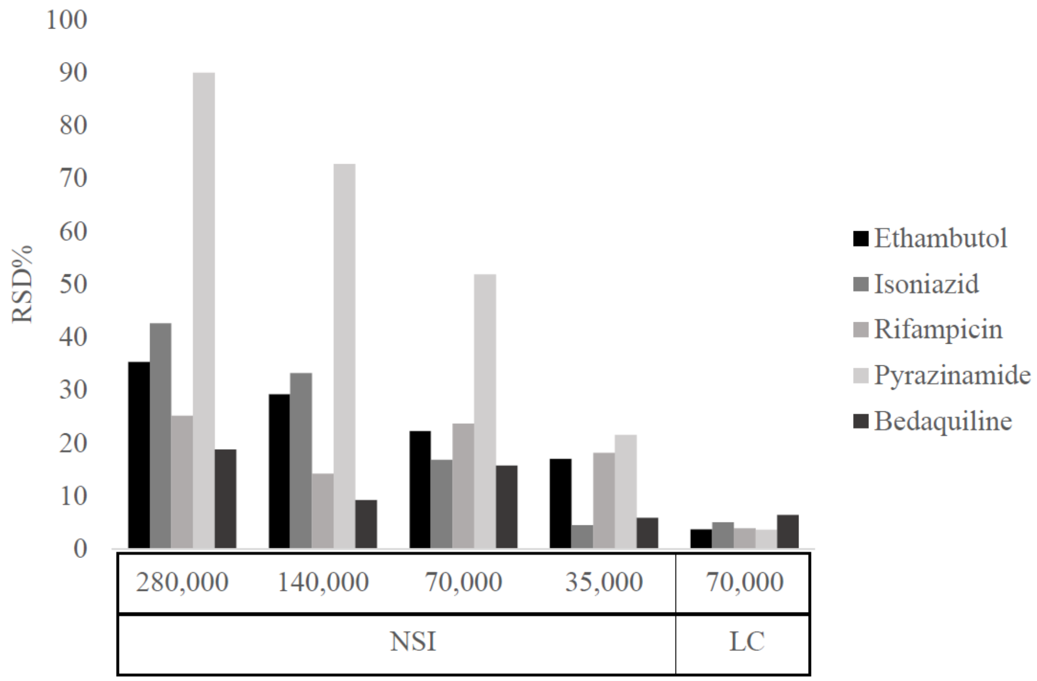

Figure S3: A graph to show the RSD% (n=5) of the [M+H]+ intensity of the five anti-TB drugs: ethambutol, isoniazid, rifampicin, pyrazinamide and bedaquiline (100 ng/ml), using NSI (3.5 minute acquisition) at four different mass resolutions: 280,000; 140,000; 70,000 and 35,000 and also LC at 70,000 (n=5) for comparison

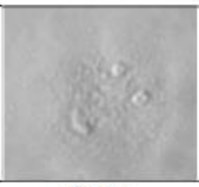	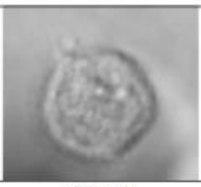	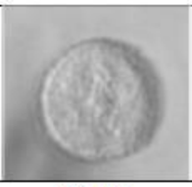	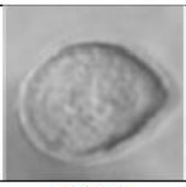	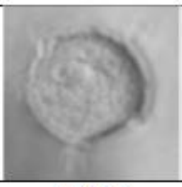
**Cell 1**	**Cell 2**	**Cell 3**	**Cell 4**	**Cell 5**
38 μm	27 μm	34 μm	35 μm	30 μm
28700 μm^2	10300 μm^2	20600 μm^2	22400 μm^2	14100 μm^2
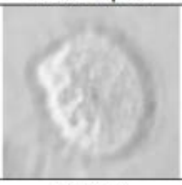	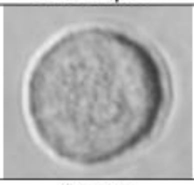	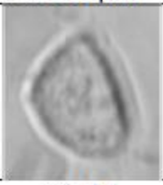	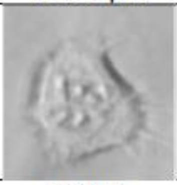	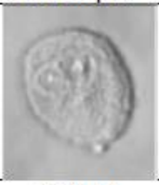
**Cell 6**	**Cell 7**	**Cell 8**	**Cell 9**	**Cell 10**
20 μm	34 μm	22 μm	35 μm	42 μm
4200 μm^2	20600 μm^2	5600 μm^2	22400 μm^2	38800 μm^2
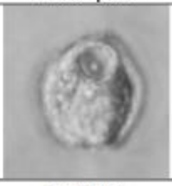	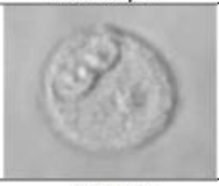	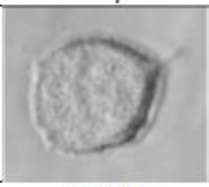	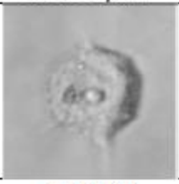	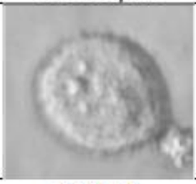
**Cell 11**	**Cell 12**	**Cell 13**	**Cell 14**	**Cell 15**
27 μm	31 μm	35 μm	27 μm	43 μm
10300 μm^2	15600 μm^2	22400 μm^2	10300 μm^2	41600 μm^2
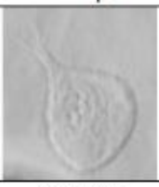	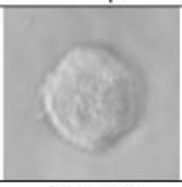	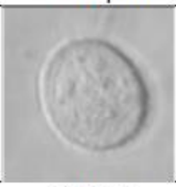	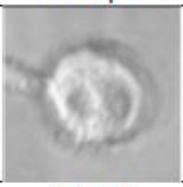	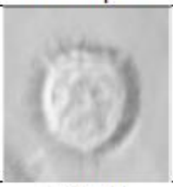
**Cell 16**	**Cell 17**	**Cell 18**	**Cell 19**	**Cell 20**
58 μm	30 μm	33 μm	27 μm	31 μm
102100 μm^2	14100 μm^2	18800 μm^2	10300 μm^2	15600 μm^2
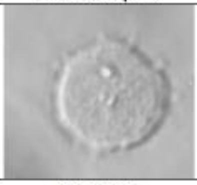	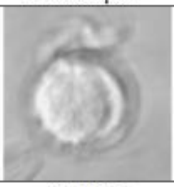	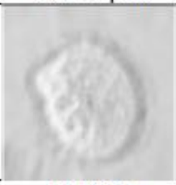	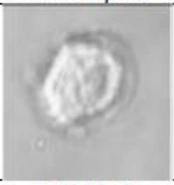	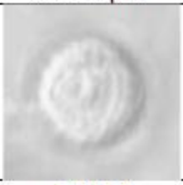
**Cell 21**	**Cell 22**	**Cell 23**	**Cell 24**	**Cell 25**
37 μm	32 μm	40 μm	24 μm	30 μm
26500 μm^2	17200 μm^2	33500 μm^2	7200 μm^2	14100 μm^2
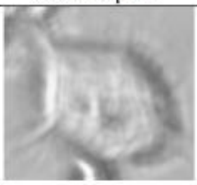	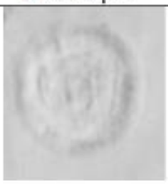	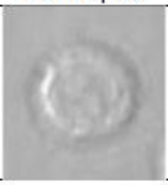	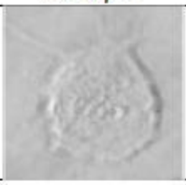	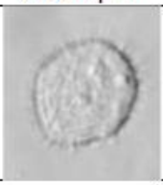
**Cell 26**	**Cell 27**	**Cell 28**	**Cell 29**	**Cell 30**
41 μm	46 μm	32 μm	51 μm	32 μm
36100 μm^2	50900 μm^2	17200 μm^2	69500 μm^2	17200 μm^2

Figure S4. Microscope images of the 30 extracted cells before nanocapillary extraction alongside the corresponding cell diameters and volumes.

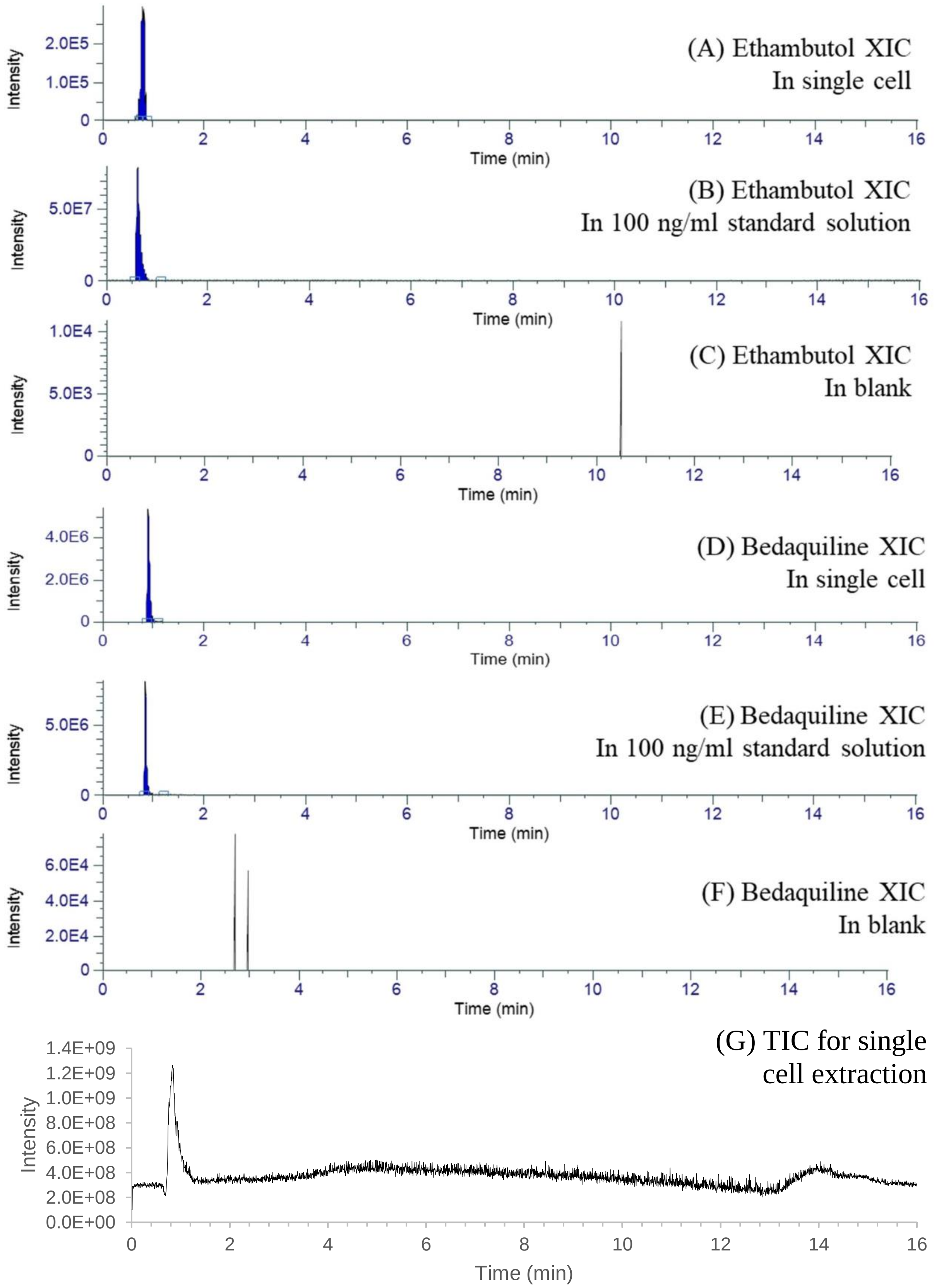

Figure S5: Extracted ion chromatograms for ethambutol (*m/z* 205.1911) in a single cell, in a 100 ng/ml standard solution and in a blank (PBS extracted using pressure injector diluted with 5µL 50:50 MeOH/EtOH solution) (A, B and C) and extracted ion chromatograms for bedaquiline (*m/z* 555.1642) in a single cell, in a 100 ng/ml standard solution and in a blank (PBS extracted using pressure injector diluted with 5µL 50:50 MeOH/EtOH solution) (D, E and F). The single cell Total Ion Chromatogram is also shown (G).

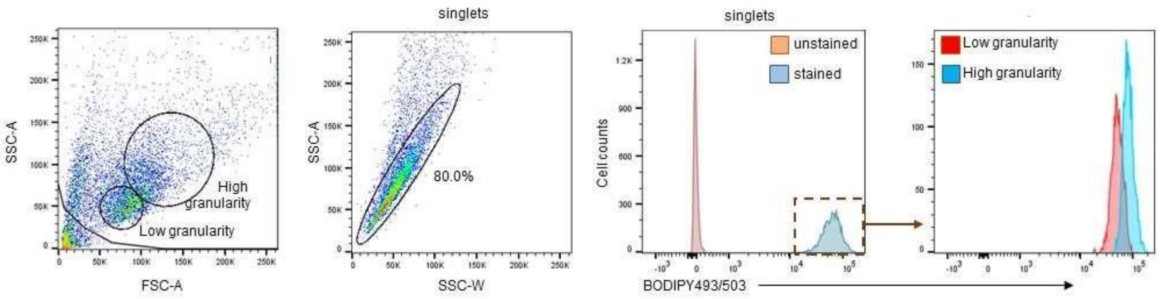

Figure S6: PANC-1 cells present high levels of lipid droplets as shown by the specific probe BODIPY 493/503.

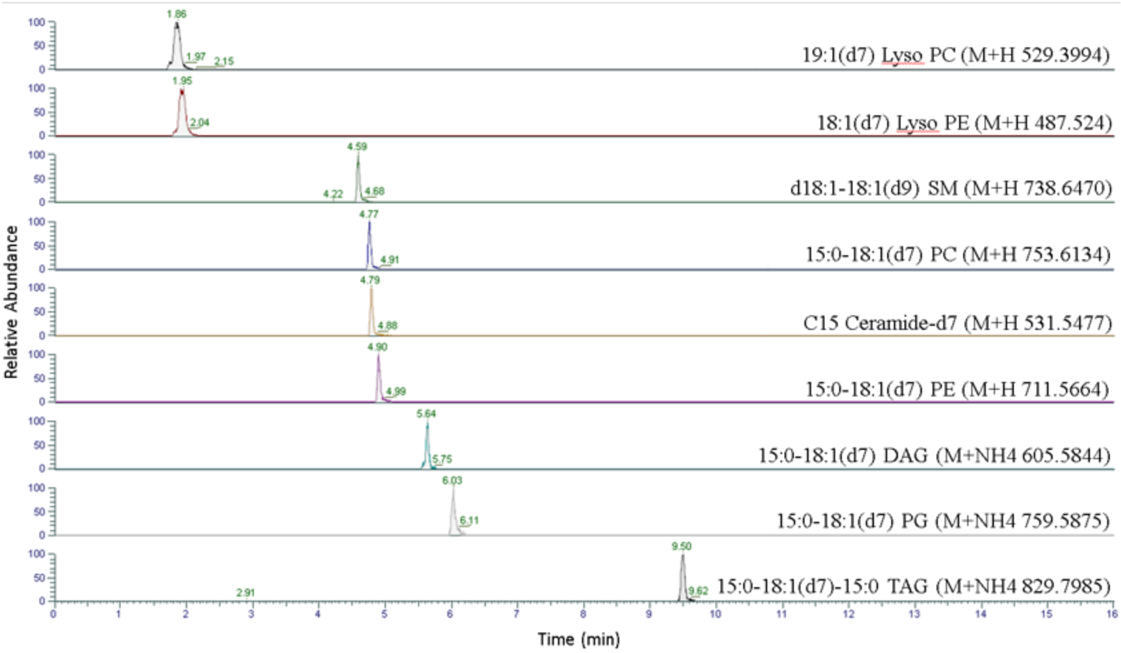

Figure S7: Extracted ion chromatograms of lipids analytes in the Avanti® Polar Lipids EquiSPLASH® diluted to 32 ng/mL dissolved in starting mobile phase, showing separation obtained using the chromatography method

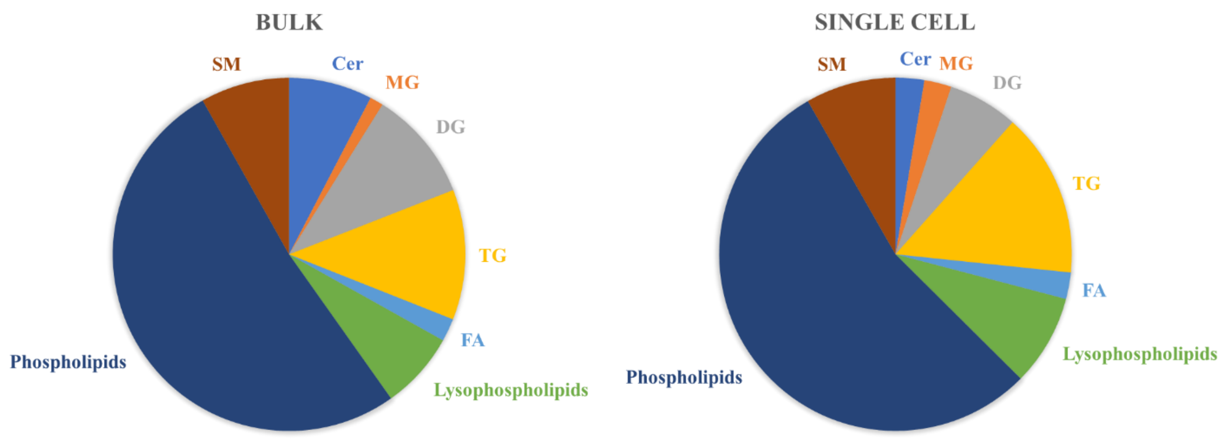

Figure S8: Charts showing proportion of assigned lipids detected (A) for cell suspension (B) averaged data for 12 single cells. Where SM: sphingolipids, Cer: ceramides, MG: monoglycerides, DG: diglycerides, TG: triglycerides, FA: fatty acids

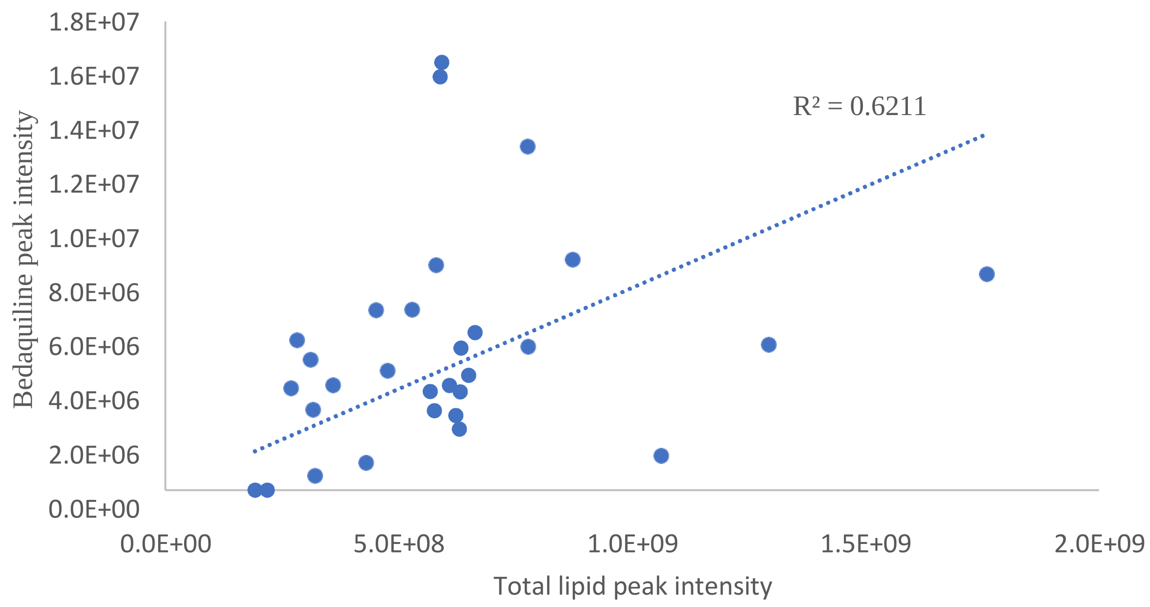

Figure S9: The total lipid peak intensity vs. the bedaquiline peak intensity for each extracted cell

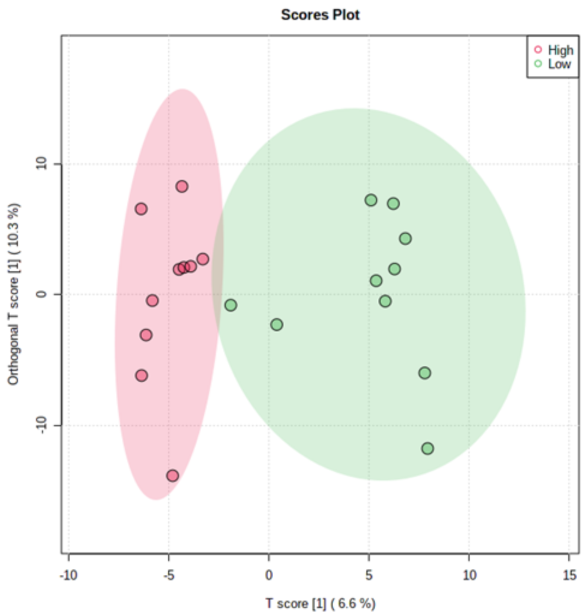

m/z	Presumptive peak assignment	VIP score
843.7412	TG(14:0e_18:2_20:4)+Li	2.23
732.5150	PC(12:0_20:4)+Li	2.18
734.5119	PC(12:1e_22:6)+H	2.18
694.6133	Cer(d20:1_26:6)+H	2.12
950.7396	PS(24:1_23:0)+Li	2.07
775.7538	OAHFA(15:0_36:1)+H	2.06
894.7134	PS(20:0e_24:1)+Li	2.00
415.3030	DG(10:0_11:3)+Li	2.00

Figure S10: (A) Partial Least Squares Discriminant Analysis (PLS-DA) for the 10 cells with the highest measured ethambutol content and 10 cells with the lowest measured ethambutol content. (B) Top variable importance in projection scores.
